# Role specialization and reproductive division of labour at the origin of eusociality

**DOI:** 10.1098/rstb.2023.0265

**Published:** 2025-03-20

**Authors:** Jeremy Field

**Affiliations:** ^1^ Centre for Ecology and Conservation, University of Exeter, Penryn, Cornwall TR10 9EZ, UK

**Keywords:** division of labour, eusociality, task specialization, subfertility, tradeoffs, life history

## Abstract

The evolution of primitive eusociality from non-social ancestors in organisms such as bees and wasps is often regarded as a major evolutionary transition. The division of labour between reproductives that specialize on egg production and workers that specialize on tasks such as foraging is the key feature defining eusociality and is why social insects are so successful ecologically. In taxa with morphological castes, individuals are often irreversibly specialized for particular roles when they reach adulthood. At the origin of sociality, however, such adaptations were absent, and we must consider why selection would favour individuals specializing when they are undifferentiated from the ancestral, non-social phenotype. Here, I focus on constraints based on life-history tradeoffs and plasticity that would be faced by ancestral females when specializing. These include limited efficiency of within-individual tradeoffs between reproductive and worker functions, imperfect matching of the productivities of social partners and lack of coordination. I also discuss the possibility that payoffs through specialization could be condition dependent. Eusocial taxa lacking morphological castes have traditionally been the testing grounds to understand the origin of eusociality, but significant adaptation has occurred since helping first evolved. Investigating role specialization at the origin of eusociality therefore requires utilizing non-social taxa.

This article is part of the theme issue ‘Division of labour as key driver of social evolution’.

## 1. Introduction

The evolution of eusociality from non-social ancestors in organisms such as bees and wasps is often thought of as one of the major evolutionary transitions, during which complex entities (social groups) are formed through cooperation between units that previously reproduced independently (individuals; [1]). A division of labour, between reproductives (or queens) that specialize on egg production and helpers (or workers) that specialize on tasks such as foraging, is the key feature defining most forms of eusociality and is also thought to be the main reason why social insects in particular are so successful ecologically. Research over recent decades has identified several genetic and ecological advantages of social nesting that should favour helping when it first arises in ancestral non-social populations. First, genetic relatedness in the ancestors of today’s eusocial Hymenoptera (wasps, bees and ants) is likely to have been high [2]. Furthermore, because females are as closely related to their siblings as to their own offspring, just a tiny ecological advantage over independent nesting should be enough to promote the evolution of daughters helping their mothers [3,4]. Second, ecological advantages of helping include insurance-based benefits to both helpers and reproductives, where a focal individual’s fitness through her reproductive effort is assured even if she dies young because nestmates will bring offspring that she only part-reared through to adulthood [5–7]. This is a potentially large advantage in Hymenoptera and could be as simple as requiring just the continued presence of nestmates, effectively guarding offspring that are already fully fed [6,8]. Improved defence against natural enemies in general, even if it occurs only incidentally because individuals are more likely to be present when they share nests, is itself often a critical advantage [9–11]. Advantages through reducing the variance in daily food provisions brought back to the nest, so that offspring are less likely to starve or face periods without food, could also be important [12], although this remains to be demonstrated convincingly.

The above advantages are quite general, should apply automatically to social groups at the origin of eusociality and have been mostly validated both empirically and in theoretical models. Yet the reality is that only a small proportion of nest-building wasp and bee species are eusocial. Why is this? Disadvantages of sociality at its origin could outweigh the advantages just mentioned. These include faster disease spread within social groups [13] (but see [14]) and the possibility that groups are actually more attractive to enemies because of the larger resource that they represent [15]. In this article, however, I will focus on a set of possible constraints based on life-history tradeoffs and plasticity that are faced by females at the origin of a division of labour and consider how these constraints might be mitigated. I also discuss the long-standing ‘subfertility’ hypothesis which postulates that the first helpers were poor-quality females with low fecundity [16]. For discussion, I assume a tradeoff-based view of life history, where an organism is assumed to have a limited set of resources, so that allocating some of them to one function necessarily reduces the quantity available for other functions. Doubts have sometimes been expressed about the validity of the tradeoff-based view [17,18], including specifically in social insects [19], but it remains a generally accepted framework.

A reproductive division of labour, and divisions of labour between worker subcastes (e.g. foragers and soldiers), is assumed to have evolved because of efficiency advantages gained through individuals specializing [20]. In ‘advanced’ eusocial insects with large colonies such as many ants, individuals are irreversibly morphologically specialized for particular tasks by the time they reach adulthood. Examples are highly fecund queens that are many times larger than their workers, and workers with extreme morphologies associated with tasks such as defence and foraging [21]. As group size increases, there is a smaller chance that any one worker will eventually replace the queen as the reproductive, so that the optimal worker phenotype becomes more specialized—for example, workers are less likely to benefit by retaining ovaries or sperm receptacles. The resulting increase in efficiency further increases group size, resulting in positive feedback with worker specialization [1,22] (see [23] for a parallel argument in humans).

When primitive eusociality first evolved in ancestral non-social taxa, however, queens and workers would have been largely or entirely undifferentiated, so that efficiency gains reliant on morphology or physiology would be minimal or absent. This raises the question that I will focus on here: whether a division of labour would initially have been favoured by natural selection. My focus will be on Hymenoptera, but I will refer to other taxa for some interesting comparisons. I will use a broad definition of ‘eusociality’, including ‘cooperative breeding’/’primitive eusociality’ (lacking obvious morphological castes) as well as ‘advanced eusociality’ with morphological castes, sometimes including irreversibly completely sterile workers (see also [24]). Note that others [25] prefer more restrictive definitions, where eusociality refers to just taxa with individuals that become irreversibly specialized as members of particular castes before they reach adulthood.

## 2. The efficiency of within-individual tradeoffs

In advanced eusocial taxa, the two castes are often highly specialized morphologically, appropriate to the tasks that they carry out. In ancestral non-social taxa, however, every female carried out all of the tasks required for reproduction, so that differentiation was minimal or absent. When a division of labour first evolves, these undifferentiated individuals switch to carrying out predominantly or entirely just one task set. A key unanswered question is therefore how efficient division of labour would be when role specialization first evolved at the origin of primitive eusociality.

Rodrigues & Kokko [26] discuss two kinds of tradeoff that could be important in social evolution: tradeoffs between individuals and tradeoffs within individuals. An example they provide of a within-individual tradeoff is whether natural selection will favour a breeder extending her longevity at a cost to her current fecundity. At the origin of eusociality, however, a critical within-individual tradeoff will be between resources required for reproductive tasks such as egg production, and those required for worker tasks such as provisioning. In the extreme, but often-imagined scenario where a mutant offspring completely takes over brood provisioning, ceding all egg-laying rights to a mother ‘queen’, the offspring could have to provision at twice the rate of an independent breeder, and the mother could have to lay twice as many eggs, to match the productivity of the ancestral strategy where the two individuals reproduced independently. Otherwise, there would be a selection on daughters favouring the ancestral strategy, unless ecological advantages (§1) were enough to compensate. Doubling each individual’s productivity in her new, specialized role might be achieved if specialization frees up time and resources no longer utilized in performing one aspect of offspring production (provisioning/egg-laying) that can then be utilized in performing the other aspect (egg-laying/provisioning). However, this would require a highly efficient tradeoff [27] between functions, well beyond the level of plasticity that ancestral non-social females are likely to have ever required. Evidence from birds suggests that such extreme specialization will be costly for ancestral taxa [28], so that partial specialization is perhaps more likely to be what evolved initially.

When it first arises, the payoff through specialization will depend on factors including the following:

- *Benefits of specialization*. These could include enhanced provisioning efficiency through (i) extra experience [29] in the worker role (e.g. learning locations of food and nests) and (ii) reduced cognitive and metabolic costs because of less frequent switching [30] between functions such as egg maturation and foraging with its associated hormonal changes [31]. If roles are adopted by females with matching phenotypes (see §5 below), efficiency could be further increased.
- *The extent to which provisioning trades off with egg production*. How much of the time and resource investment associated ancestrally with each role (e.g. ovary development) is irreversibly committed, and how much is transferable to the alternate role (e.g. to fuel foraging)? In insects with dispersal polymorphisms, dispersive morphs with larger flight muscles have smaller ovaries and lower fecundity than non-dispersive morphs, with the maintenance as well as production of flight muscles competing for nutrients with egg production [32]. It is clear that investment patterns can change dramatically during evolution—for example, some ant workers completely lack ovaries and sperm storage organs. But how plastic resource allocation would be at the origin of role specialization is unknown. Ancestral taxa are likely to possess some evolved plasticity in response to natural variation in factors such as scarcity of provisions (influencing the amount of foraging required to feed offspring) or nest density (facilitating conspecific egg replacement and hence opportunities to oviposit [33]). It might also be possible to acquire additional resources required for extra egg production or foraging through adult feeding, although this may come with extra risks such as predation [32].
- *The existence of environmentally or physiologically imposed limits*. The ability of females newly in the worker role to increase their provisioning rates, even if there is pre-existing plasticity in resource allocation, could be constrained by factors such as fixed hours of daylight and accumulating damage through processes such as unavoidable wing wear [34] and oxidative stress [35]. Increased provisioning would also directly increase a proto-worker’s mortality through predation away from the safety of the nest. For females newly in the reproductive role, eggs are relatively large and costly, and females of non-social taxa typically have only one or two mature oocytes in their ovaries at any one time [36]. There is therefore likely to be a limit on short-term rates of egg production as well as lifetime fecundity in newly specialized reproductives. However, widespread intraspecific parasitism in today’s non-social taxa, where females lay eggs opportunistically in each other’s nests [33], implies that at least some spare egg-laying capacity is present.

## 3. Complementarity

A related issue potentially reducing the efficiency of a division of labour when it first evolves is imperfect matching in terms of the productivities of social partners when they specialize. To take a concrete example, Field *et al*. [37] estimated that the parental investment costs spent by a female solitary digger wasp in producing an egg were approximately half of those required to provision the resulting offspring. Ignoring for a moment other potential costs of offspring production, this implies that one-third of resources are spent on eggs and the other two-thirds on provisioning. If resources were fully transferable between roles, a female that starts to specialize entirely as a reproductive should, therefore, be able to treble her egg production by utilizing the resources she previously used for provisioning. However, a specialist worker could increase her provisioning by only 50% using resources freed up by not producing eggs. In a social group comprising one female of each type, offspring will therefore be underfed, or some eggs will be wasted. The reverse problem can operate in models of social evolution, when groups get too large for a single queen’s fecundity to keep up with the provisioning potential of her helpers [38]. A solution in both cases could be a greater number of individuals performing the costlier tasks in the first social groups, or incomplete specialization by the partner with the less costly task set. In the digger wasp example above, this could equate to groups comprising two specialist helpers that should together be able to feed the offspring produced by a specialist reproductive; or a specialized helper nesting with a less specialized reproductive that also carries out some of the provisioning. Cooper & West [39] found that the latter was never an endpoint in their model of how a division of labour evolves, but it might represent an intermediate state and is one that can occur in small groups of paper wasp co-foundresses [40]. In some taxa, provisioning is probably less costly relative to egg production, for example, when individual prey items are smaller and require less effort to carry back to the nest, or when provisions are tiny pollen grains so that load size can be matched to an individual’s load-carrying capability [41–43]. The productivities of specialist helpers and reproductives should then be better matched.

## 4. Coordination

In non-social wasps and bees, mothers provision a series of offspring that leave to initiate their own nests when they reach adulthood. Interactions between long-lived mothers and their newly matured offspring, and between offspring themselves, may lead to an increase in tolerance of relatives at the nest. The origin of a division of labour between mother and offspring might then involve a mutation that causes offspring to forego their own reproduction and help provision further siblings (their mother’s offspring); or a mutation that causes the mother to stop foraging for provisions when her first offspring mature as adults, to instead focus on egg-laying. However, sociality is by definition not a property of individuals, and an obvious constraint that could reduce the efficiency of reproduction in mutant nests is a lack of coordination: offspring might not have the behavioural plasticity required to provision eggs provided by mutant mothers, even if it is in their fitness interests to do so, and might continue to oviposit themselves. Similarly, mothers might not accept provisions collected by their mutant offspring, or might not compensate by reducing their own provisioning effort (‘load-lightening’). The latter might seem unlikely, but at nests of a non-social apoid wasp, mothers appeared to provide a fixed quantity of food to each offspring rather than directly assessing how much food was already present: they failed to compensate by adding extra food when some of the food was removed experimentally [44].

Elgar & Jebb’s [44] experiment involved a so-called mass provisioning wasp where all of the provisions are placed in the offspring cell before the egg has even hatched, and mothers never encounter their larvae. In birds, parents adjust their provisioning effort in response to begging cues from growing offspring [45]. Compensation might therefore be more likely in progressively provisioning wasps and bees that feed offspring more gradually as they grow, perhaps involving continuous assessment of offspring needs (as in some eusocial taxa [46,47]). This could potentially help to explain the positive association between progressive provisioning and eusociality [48]. Indeed, a similar experiment with the progressively provisioning sand wasp *Ammophila* suggested that females do compensate at least partially when provisions are removed experimentally [37]. However, a set of observations that relate more directly to a helping scenario produced a different result. Two different *Ammophila* females sometimes provisioned the same offspring simultaneously after one or both had carried out egg replacements (within-species brood parasitism). Each female then provided the same amount of food as a lone mother, and resulting offspring were correspondingly heavier [49]. This apparent lack of compensation seems likely to be maladaptive if the relationship between total provisions and offspring fitness is convex, so that extra food would have been better utilized feeding extra offspring [50]. Why would joint-provisioning mothers fail to compensate by reducing their effort? One possibility is that if there is a significant chance of offspring starvation, the extra food might not in fact be maladaptive [51]. However, starvation is unlikely to be an important issue for non-social wasps and bees [52], and a simpler hypothesis is that mothers use their own provisioning effort as a rule-of-thumb measure of food provided.

The occurrence of compensation has been little investigated in wasps and bees, and it is possible that in lineages where eusociality has evolved, ancestral non-social taxa used a different rule of thumb that did lead to compensation. Alternatively, if the first step towards eusociality involved one female specializing as a reproductive, perhaps with multiple immatures available to provision simultaneously, compensation may have been unnecessary. Occasional nest sharing has been reported in many species that are normally non-social (e.g. see the compilation in [9]), providing material ripe for investigation.

The lack of coordination in *Ammophila* contrasts somewhat with experiments on ant queens that normally initiate nests alone and non-social species of sweat bees and carpenter bees. Interestingly, when forced to nest together, females in these taxa divided tasks with each other to some extent, so that a division of labour appeared to be an emergent effect of grouping [53–55]. This could mean that some lineages are intrinsically predisposed to enjoy greater efficiency when social nesting first evolves. However, care is needed with this interpretation, as these are taxa that are eusocial in other phases of their life cycles (ants), have evolved from eusocial ancestors (sweat bees [56]), and/or sometimes nest socially so that the social phenotype will have been exposed to selection (sweat bees, carpenter bees). The coordination observed may have evolved only after the origin of sociality.

Coordination may be less of a constraint in other major eusocial/cooperatively breeding lineages. In some vertebrates such as birds, non-cooperatively breeding sister lineages are usually pair breeders, with both sexes provisioning the offspring to some extent [57]. Similarly, some non-cooperatively breeding cichlid fishes are pair-breeders [58]. Unlike non-social Hymenoptera, where a single mother provisions her offspring, such pair-breeding vertebrates will already have evolved adaptive responses to provisioning by social partners, using rules of thumb that could be coopted in cooperative breeding: efficient coordination at the origin of cooperative breeding may have been less problematic. In taxa such as wood-inhabiting lower termites or cooperatively breeding ambrosia beetles [59,60] where offspring feed themselves on the nesting substrate and/or cultivated fungi, so that parental care does not require collecting provisions away from the nest, coordination between adults may be less of an issue in general.

## 5. Condition dependence

In birds, cooperation is often thought to represent a ‘best-of-a-bad-job’ strategy, where offspring face high mortality risks by dispersing, or a lack of food, mates and/or nesting sites [61]. A related, long-standing hypothesis in the field of insect sociality is that the first helpers were females with low reproductive value [16,62]. Specifically, ‘subfertile’ females with low potential fecundity might forfeit little by helping fully fertile relatives. Given that fecundity tends to be correlated with body size in insects [63], subfertility might equate with small size. Because mothers can double their genetic representation by replacing grandchildren with offspring, selection might even have favoured maternal manipulation of daughter phenotypes so that a daughter’s best option was to help (see table 1) [4,64–66]. However, maternal manipulation [67] (and condition-dependence) typically cannot logically evolve before helping itself, since manipulated offspring must have the option of responding to their phenotypes by helping (see [67] for a related discussion about the origin of morphologically specialized castes).

**Table 1.** Hypothetical productivities (left-hand three columns; number of offspring) and logical preferences (columns 4−5) for a daughter that has the choice between helping her singly mated mother reproduce (e.g. by foraging for provisions to feed the mother’s offspring) versus reproducing independently herself (the first of these choices represents the ‘subsocial’ route through which eusociality appears to have evolved in the Hymenoptera [2]). The top row exemplifies the classic subfertility scenario, while the other rows represent related scenarios discussed in the main text.

productivities (no. offspring)			preferences		
**daughter alone**	**mother alone**	**mother with helper**	**daughter** (**IF alone, IF helping**)	**mother (IF alone, IF with helper**)	**scenario**
2.5	10	15	help (1.25,2.5)	help (0.625,2.5)	classic ‘Subfertility’
5	10	15	indifferent (2.5,2.5)	help (1.25,2.5)	daughter both sub-fertile and sub-forager
5	10	12	alone (2.5, 1)	alone (1.25, 1)	daughter sub-fertile but more strongly sub-forager

IF = inclusive fitness with relatedness = 0.5 between daughters and their siblings or their own offspring. For example, in the first row daughters produce fewer offspring nesting alone than mothers, but also fewer than they can add to their mother’s brood as helpers: if a daughter chooses to nest alone, 1.25 copies of her genes are produced through her 2.5 own offspring. If she instead helps her mother, she obtains 2.5 offspring equivalents, entirely through her mother. She should therefore prefer to help. In the second row, although daughters are less productive than their mothers when nesting alone, they are just as unproductive in the helper role, perhaps because they are of generally low quality, or because mothers have limited fecundity. In the third row, daughters are even less productive when helping than when nesting alone. Note that from the mother’s perspective, we are comparing a daughter’s decisions in terms of the mother’s IF at a fixed ecological time step (the time step when the daughter has either helped her mother produce offspring, or nested alone to produce her mother’s grandchildren). Note also that all else being equal, a mother that had the choice between producing two ‘subfertile’ daughters in row one or one daughter in row 2 should choose the former if doing so makes it more likely that daughters choose to help. For simplicity, the table excludes possible effects on behaviour of other daughters

Selection could similarly favour certain phenotypes in the reproductive role (e.g. subfertile females helping only highly fecund recipients that stand to gain large benefits from their help [62]). More generally, particular combinations of phenotypes might be especially complementary, allowing specialization to evolve more easily [68]. Size and fecundity are not the only traits that could affect the costs and benefits of specialization. For example, there is evidence that worker bees with more antennal olfactory receptors carry out more foraging for offspring provisions [69].

A critical untested assumption of the subfertility hypothesis concerns the provisioning ability of small/subfertile females. Such females may have low fecundity when nesting independently, but the subfertility hypothesis assumes that they will perform disproportionately better at worker tasks such as provisioning, so that adopting a helper role is their optimum strategy (table 1, first row). Low-quality females might instead perform particularly badly when provisioning (table 1, third row), or perform equally poorly at all tasks (table 1, second row), so that helping is not particularly favoured [16].

Note that the finding, in many non-social nest-building wasps and bees, that smaller females produce offspring at lower rates [43,70], does not necessarily mean that small females will be poor provisioners as well as having low fecundity. Because different components of care are inter-dependent (e.g. provisioning necessarily requires an egg to have been laid), it is unclear whether females of particular sizes perform worse with some components than others. For example, small females might be capable of provisioning at a higher rate than observed, but be unable to express this in nature because of constrained fecundity. However, observational studies of life history in other taxa typically do find that low-quality individuals perform consistently badly across numerous traits [17,28]). Furthermore, the pattern discussed in the previous section, where the aspects of parental care undertaken by workers are more costly than egg production, could make the subfertility hypothesis even less likely to operate. However, if helping is less costly for higher quality individuals [71], the opposite pattern might be favoured, where higher quality individuals help lower quality individuals that are still capable of producing enough eggs for the former to feed.

Are there situations where it seems more likely *a priori* that subfertility might operate than others? One such situation could be where fecundity is phenotype-dependent, but species ecology means that helping is valuable independent of phenotype. An example might be where helpers are primarily guards defending the nest against smaller heterospecific enemies such as ants or some parasitoids, so that defensive ability is not phenotype dependent. Another scenario would be where some females, while still being capable of provisioning, have low fecundities because of their physiologies, such as when they are effectively castrated by parasites [72]. In some empirical studies of social insect workers, foraging ability does appear to be independent of size [64]. If the positive relationship between size and fecundity still holds, the payoff through helping would be relatively higher for smaller females.

## 6. Discussion

The elaboration of social behaviour after group formation has evolved is thought to require so-called tradeoff-breaking adaptations that enable the group to function more efficiently than its original parts (the constituent individuals [73]). These adaptations, which include the morphological specializations seen in large eusocial groups, make the individuals within the group less able to function independently. At the extreme where each queen or worker is unable to perform the alternative role and no longer commits resources to it, such adaptations could remove or minimize the within-individual tradeoff between reproductive and non-reproductive tasks discussed above [74]. At the origin of sociality, however, such adaptations were absent, and we have to consider how and why selection favours individuals specializing for particular roles when they are largely undifferentiated from the ancestral, non-social phenotype. An important goal for the relatively mature field of social evolution is to understand why eusociality is a relatively rare strategy, evolving and diversifying strikingly in some lineages while other speciose and apparently biologically similar lineages comprise entirely non-social taxa. Variation in genetic relatedness or in the balance between ecological costs (e.g. disease spread) and benefits (e.g. defence) are two likely contributors, but here I have highlighted intrinsic life-history features as another. Variation between lineages in terms of the efficiency and plasticity of life-history tradeoffs between queen and worker functions, in terms of whether those tradeoffs depend on phenotype, and in terms of how the mechanisms involved in provisioning will impact coordination could critically affect payoffs obtained at the origin of the role specialization that is the key feature of eusociality. In some lineages, these factors could represent a fitness valley which makes specialization unlikely to evolve. In other lineages this may not be the case, for example, if ancestral mothers had excess eggs that could potentially be provisioned by helpers, or where egg production and provisioning have similar costs, so that a specialist helper can potentially feed all of the offspring produced by a specialist reproductive. Alternatively, life history-based constraints could be outweighed by ecological benefits, and where helping takes the form of guarding the nest against enemies that can be repelled independent of phenotype, females with low fecundity could be disproportionately effective as helpers.

Primitively eusocial wasps and bees (lacking morphological castes) have traditionally been the main testing grounds to understand the origin of eusociality in Hymenoptera. However, significant adaptation has occurred in the more than 20 Myr [75,76] since helping evolved, and both obligate [77,78] and facultatively [79] primitively eusocial taxa indeed exhibit numerous derived features such as sophisticated dominance hierarchies [77], role plasticity [78] and social signalling [80]. For example, in today’s primitively eusocial taxa, the number of offspring reared typically increases in proportion with the number of workers [81], maintaining *per capita* productivity. It is unclear whether this would be the case at the origin of eusociality. Why would a female from a previously solitary taxon have already evolved the ability to double or treble her fecundity, as required for an efficient division of labour when she becomes a ‘proto-queen’? Investigating role specialization at the origin of eusociality therefore also requires utilizing non-social taxa, where adaptations to social living are reduced or absent. Experimental approaches could include: (i) estimating the potential fecundity of non-social females as specialist reproductives, and its relationship with phenotype, by repeatedly removing newly-laid eggs; (ii) estimating how closely the costs of offspring provisioning match those of egg production [37]; or (iii) testing whether non-social females will accept provisions taken from conspecifics and added to nests experimentally [82], and how they adjust their own level of offspring provisioning in response [49].

Care is needed in selecting specific non-social taxa as proxies for the ancestors of eusocial relatives. First, today’s non-social taxa will have traits that have been derived since the last common ancestor with eusocial taxa [83]. Second, the apparent homogeneity of nesting biologies among non-social Hymenoptera may mask important life-history variation. In cooperatively and communally breeding birds, for example, there is a range of responses to helper presence, from species where breeders maintain their provisioning effort so that more food is provided per offspring, to load-lightening where food availability is unchanged, to larger brood sizes in the presence of helpers where food availability per offspring may decrease [84]. Tractability allowing, the proxy ancestor would therefore ideally be a sister clade that is as close phylogenetically to a focal eusocial lineage as possible (see [85]). In the Hymenoptera, such taxa include potter wasps (Eumeninae), the sister group of eusocial vespines and polistines [86]; and various clades of apoid wasps and bees which represent the more or less distant sister clades of eusocial bees and silk wasps (*Microstigmus* [56,87,88]). In comparison with vertebrates, where the pair-breeding sister taxa of cooperative breeders are relatively well studied, the non-social sister taxa of eusocial insects are much less well understood, so that the above discussion is necessarily speculative. In particular, there is no research body measuring the fundamental life-history tradeoffs present in non-social insects that compares with the numerous experimental studies that have been carried out on taxa such as birds [17,28]. Measuring these tradeoffs has the potential to shed significant light on the uneven distribution of eusociality among insect lineages.

## Data Availability

This article has no additional data.
